# The Acquired Vulnerability Caused by CDK4/6 Inhibition Promotes Drug Synergism Between Oxaliplatin and Palbociclib in Cholangiocarcinoma

**DOI:** 10.3389/fonc.2022.877194

**Published:** 2022-05-17

**Authors:** Orawan Suppramote, Sunisa Prasopporn, Satinee Aroonpruksakul, Ben Ponvilawan, Jiradej Makjaroen, Monthira Suntiparpluacha, Krittiya Korphaisarn, Komgrid Charngkaew, Rawisak Chanwat, Trairak Pisitkun, Seiji Okada, Somponnat Sampattavanich, Siwanon Jirawatnotai

**Affiliations:** ^1^ Siriraj Center of Research Excellence (SiCORE) for Systems Pharmacology, Department of Pharmacology, Faculty of Medicine Siriraj Hospital, Mahidol University, Bangkok, Thailand; ^2^ Princess Srisavangavadhana College of Medicine, Chulabhorn Royal Academy, Bangkok, Thailand; ^3^ Center of Excellence in Systems Biology, Faculty of Medicine, Chulalongkorn University, Bangkok, Thailand; ^4^ Division of Medical Oncology, Department of Medicine, Faculty of Medicine Siriraj Hospital, Mahidol University, Bangkok, Thailand; ^5^ Department of Pathology, Faculty of Medicine Siriraj Hospital, Mahidol University, Bangkok, Thailand; ^6^ Hepato-Pancreato-Biliary Surgery Unit, Department of Surgical Oncology, National Cancer Institute, Bangkok, Thailand; ^7^ Division of Hematopoiesis, Joint Research Center for Human Retrovirus Infection, Kumamoto University, Kumamoto, Japan

**Keywords:** acquired vulnerability, ribosomal biogenesis stress, CDK4/6 inhibitor, oxaliplatin, cholangiocarcinoma (CCA), palbociclib

## Abstract

Cholangiocarcinoma (CCA) is one of the most difficult to treat cancers, and its nature of being largely refractory to most, if not all, current treatments results in generally poor prognosis and high mortality. Efficacious alternative therapies that can be used ubiquitously are urgently needed. Using acquired vulnerability screening, we observed that CCA cells that reprofile and proliferate under CDK4/6 inhibition became vulnerable to ribosomal biogenesis stress and hypersensitive to the anti-ribosome chemotherapy oxaliplatin. CCA cells overexpress the oncogenic ribosomal protein RPL29 under CDK4/6 inhibition in a manner that correlated with CDK4/6 inhibitor resistance. Depletion of RPL29 by small interfering RNAs (siRNAs) restored the sensitivity of CCA cells to CDK4/6 inhibition. Oxaliplatin treatment suppressed the RPL29 expression in the CDK4/6 inhibitor treated CCA cells and triggered RPL5/11-MDM2-dependent p53 activation and cancer apoptosis. In addition, we found that combination treatment with oxaliplatin and the CDK4/6 inhibitor palbociclib synergistically inhibited both parental and CDK4/6 inhibitor-resistant CCA, and prevented the emergence of CDK4/6 and oxaliplatin-resistant CCA. This drug combination also exerted suppressive and apoptosis effects on CCA in the *in vitro* 3-dimensional culture, patient-derived organoid, and *in vivo* xenograft CCA models. These results suggest the combination of the CDK4/6 inhibitor palbociclib and the anti-ribosome drug oxaliplatin as a potentially promising treatment for cholangiocarcinoma.

## Introduction

Cholangiocarcinoma (CCA) is a highly lethal gastrointestinal malignancy that has one of the worst prognoses among solid tumors. Most patients with CCA are diagnosed at a late stage. Although, surgery is the only curative treatment; most patients are ineligible for tumor resection. Gemcitabine plus cisplatin is recommended as first-line treatment for advanced stage disease with objective response rate only 25 percent (1).

Although novel molecular targetable mutations, such as activating IDH1/2 mutations and FGFR fusions, have been identified, these mutations present in only a small percentage of patients (2). Almost all patients eventually experience disease progression while on first-line systemic therapy and succumb to their disease. Therefore, novel therapeutic drugs/strategies to treat CCA are urgently needed. Targeted therapies that can be broadly applied do not yet exist, because CCA includes genetically heterogeneous tumors with no single major molecular driver (3–8).

Previously reported evidence showed that deregulation of genes that encode cell cycle regulators is common in CCA, which highlights cell cycle inhibition as a logical strategy for CCA treatment (9), and several preclinical studies, including our work, have reported corroborating evidence (10–12). However, a clinical study in CDK4/6 inhibitor monotherapy for CCA fell short of expectations (13). There are some hypotheses that may explain the aforementioned disappointing results. For one thing, the shortcoming in clinical level may be a result of a failure to identify the CCA with suitable molecular profiles for the CDK4/6 inhibition. Second, the efficacy of CDK4/6 inhibitor monotherapy may be compromised by the emergence of acquired drug resistance, which can develop rapidly. These challenges will need to be overcome to leverage the benefit of the cell cycle inhibitors in CCA. Considering the inevitable emergence of drug resistance, identification of rational combinations is an appealing concept, and one of the most active fields of study in cancer drug resistance (14).

It has been demonstrated that cancer cells that are being treated with drugs try to adapt and reprofile their molecular networks to survive, proliferate, and become drug-resistant. This adaptation comes at a fitness cost of some collateral physical characters, which may result in an acquired vulnerability (acquired sensitivity) within the drug-resistant cancer cell (15).

In the present study, we hypothesized that while under strong CDK4/6 inhibition, CCA cells would reprofile their molecular networks and, as a result, become dependent on a new converged biological process/pathway to survive the inhibition. We then set forth to identify the newly emerging therapeutic targets in CDK4/6 inhibitor-resistant cancer cells by leveraging the acquired vulnerability of the cancer cells that grew under CDK4/6 inhibition in the hope that inhibition of the novel target would synergize with CDK4/6 inhibition and prohibit the escape of CCA from CDK4/6 inhibition. We performed acquired vulnerability screening and analyzed pathway reprofiling in CDK4/6 inhibitor-resistant CCA. Our results revealed an acquired vulnerability of resistant CCA cells to ribosome biogenesis stress, and drug synergism between CDK4/6 inhibitor and a drug already used in the treatment of CCA, oxaliplatin. We also uncovered the mechanistic basis for the observed drug synergism, and validated the efficacy of this drug combination in both *in vitro* and *in vivo* CCA models, which facilitates direct translation for clinical investigation of these findings.

## Materials and Methods

### Cell Culture and the Establishment of Resistant Cell Lines

KKU-055 and KKU-213B cell lines were obtained from the Japanese Collection of Research Bioresources (JCRB) Cell Bank (Osaka, Japan). The culture methods were previously described (10). Cell lines were routinely tested for mycoplasma. Palbociclib-resistant cell lines and single-resistant clones were generated as previously described (16, 17).

### Cell Viability Assay

Palbociclib, ribociclib, abemaciclib, oxaliplatin, cisplatin, and actinomycin D were purchased from Selleck Chemicals (Houston, TX, USA). Phenanthriplatin was generously provided by Professor Stephen J Lippard of the Department of Chemistry, Massachusetts Institute of Technology, Boston, MA, USA. Drug testing in resistant cell lines and 3-demensional (3D) spheroids was performed as previously described (10).

### Western Blotting and Immunoprecipitation Assay

The whole-cell lysates were prepared by lysis in 0.5% NP-40 buffer (NP-40, 1 M HEPES pH 7.4, 5M NaCl, 0.5M EDTA pH 8) supplemented with a protease inhibitor and a phosphatase inhibitor (Thermo, 78420, USA). The nuclear-cytoplasmic fractionation lysates were prepared using cytoplasmic buffer (10 mM HEPES, 1.5 mM MgCl2, 10 mM KCl, 0.5 mM DTT, 0.05% NP-40, pH 7.9) and nucleoplasmic buffer (5 mM HEPES, 1.5 mM MgCl2, 0.2 mM EDTA, 0.5 mM DTT, 26% glycerol, pH 7.9). Equal amounts of lysate were used for Western blotting as previously described (10). Immunoprecipitation was performed according to standard protocol (18).

### Immunofluorescence Staining

Cells were seeded in six-well plates at a 2,000 cell/well density and incubated for 24 hours. The next day, the culture medium was removed and new culture medium treated with 5 μM oxaliplatin, 0.5 μM cisplatin, or Vehicle was added into different wells and reincubated for 24 hours (10).

### Proteomic Analysis by Quantitative Mass Spectrometry

Two palbociclib-resistant clones (KKU-055R29 and KKU-055R30) and the KKU-055wt were selected for 3-plex dimethyl labeling proteomic analysis. The cells were lysed in 8 M urea lysis buffer containing protease and phosphatase inhibitors (Thermo, 78420, USA). Equal amounts of whole-cell lysate were digested, labeled, fractionated, and subjected to liquid chromatography-tandem mass spectrometry (LC-MS/MS) using the Q Exactive™ Plus Hybrid Quadrupole-Orbitrap™ mass spectrometer as previously described (19). The raw data were analyzed for protein identification and relative quantification using MaxQuant software (Max Planck Institute of Biochemistry, Planegg, Germany). The files were searched against the human UniProt database (August 2019) using previously described search parameters (19). Proteins matched the reverse database and contaminants were excluded, and imputed missing values were derived from the normal distribution (width: 0.3, downshift: 0.5). The pathway analysis was performed and the statistics were calculated using Perseus software (version 1.6.14.0, Max Planck Institute of Biochemistry).

### Gene Set Enrichment Analysis

Gene expression profile from the Kyoto Encyclopedia of Genes and Genomes (KEGG) ribosome signature gene set (88 genes by gene family) analysis was performed as previously described (10). The normalized enrichment score was calculated, and the genes with a positive rank metric score were specified.

### *In Vivo* Studies

The protocols for all *in vivo* experiments in this study were approved by the Mahidol University – Institute Animal Care and Use Committee. Experiments were performed as previously described (10). A total of 10 × 10^6^ of KKU-055 cells was subcutaneously injected into female 2-month-old non-obese diabetic/severe combined immunodeficiency (NOD/SCID) mice, and the resulting tumor was allowed to grow to 0.5 cm in diameter. After that, 75 mg/kg palbociclib was administered daily *via* oral gavage until the tumor re-grew during treatment to obtain a palbociclib-resistant tumor. The palbociclib-resistant tumor was then excised and engrafted into a new cohort of study mice. After confirmation of tumor engraftment, 75 mg/kg palbociclib daily *via* oral gavage, 5 mg/kg oxaliplatin weekly *via* intraperitoneal injection, or the combination of both was given to tumor engrafted study mice. Hematoxylin and eosin (H&E) staining of mouse tumor tissues was performed according to standard protocol (10).

### Drug Testing in Patient-Derived Organoids

The study protocol was approved by the Institutional Review Board for Human Research (SI494/2019 and NCI006/2020). Intrahepatic CCA tissues were pasted using surgical blades and then washed with 12 ml of wash media (advanced DMEM/F12 containing 1 x Glutamax™ (Thermo, 10565018, USA), 10 mM HEPES, and 100 U/ml Penicillin/Streptomycin). The tissue paste was collected by centrifugation at 400 x g, 4°C for 5 min, then digested with 5 ml of the wash media containing 2 mg/ml collagenase D at 37°C for 30 min. Undigested tissues were filtered out with cell strainers. The cells were embedded in 70% matrigel and cultured in organoid culture media (20) until organoids formed. The organoids were dissociated into single cells by incubating with 1 ml of TrypLE^TM^ Express (Gibco, 12604021, USA) at 37°C for 5 min. In a 384-well plate format, the cells suspended in organoid culture media containing 5% matrigel were plated at 1000 cells/well, and incubated for 72 h. Eight concentrations of each drug with the following final concentrations: Palbociclib at 10, 5, 1, 0.5, 0.25, 0.125, 0.062, and 0.031 mM; Oxaliplatin at 100, 50, 10, 5, 2.5, 1.25, 0.62, and 0.31 mM; Palbociclib plus Oxaliplatin combination at 1:1 ratio, were added and incubated for 5 days. Cell viability was measured using ATPlite™ Luminescence Assay System (Perkin Elmer, Waltham, MA, USA) following the manufacturer’s instruction, and the cell viability percentages were calculated by normalizing with non-treated control (0.05% DMSO).

### Statistical Analysis

Unless otherwise stated, comparison was made and statistical significance was determined between groups using a two-sided Student’s t-test. A p-value of less than 0.05 was considered statistically significant for all tests. Data are shown as the mean ± standard deviation (SD) of at least 3 experiments. Analysis of variance (ANOVA) or Kruskal-Wallis test was used for comparisons among 3 or more groups.

## Results

### Characterization of CDK4/6 Inhibitor-Resistant CCA Cells

**Figure 1:**
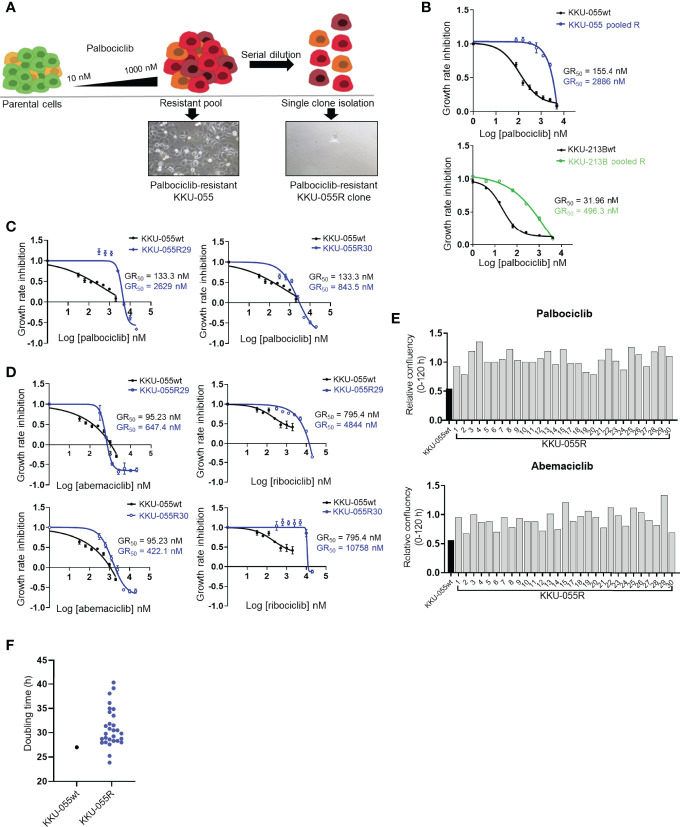
Characterization of CDK4/6 inhibitor-resistant CCA cells. **(A)** Palbociclib-resistant KKU-055 and KKU-213B CCA cells (KKU-055 pooled R, and KKU-213B pooled R) were generated using a step-wise dosing protocol, and then isolated into single clones *via* serial dilution. All resistant cells were maintained in 1 µM palbociclib. **(B)** Palbociclib dose-response curves for parental KKU-055wt and pooled resistant cells. Error bars represent standard deviation of triplicate cultures. **(C)** Palbociclib dose-response curves for KKU-055wt and resistant clones. Error bars represent standard deviation of triplicate cultures. **(D)** Abemaciclib and ribociclib dose-response curves for KKU-055wt and resistant clones. Error bars represent standard deviation of triplicate cultures. **(E)** Relative confluencies of resistant KKU-055 clones compared to KKU055wt under 140 nM palbociclib or 100 nM abemaciclib treatment at 120 hours. **(F)** Doubling time of KKU-055wt and twenty-nine KKU-055-resistant clones.

To investigate acquired vulnerability in CCA cells that proliferate despite CDK4/6 inhibition, we developed the CDK4/6 inhibitor-resistant CCA cell lines KKU-055R and KKU-213BR from parental KKU-055 (KKU-055wt) and KKU-213B (KKU-213Bwt) cells by culturing the parental cells with the CDK4/6 inhibitor palbociclib using a step-wise dosing protocol (**Figure 1A**). The KKU-055R and KKU-213BR palbociclib GR50s were 18.57- and 15.53-fold higher than that of the parental cells, respectively (**Figure 1B**). We isolated and expanded up to 29 single cells (clones) from KKU-055R, and 13 clones from KKU-213BR to study various mechanisms of CDK4/6 inhibitor resistance (**Figure 1A**). We validated that these drug-resistant clones were resistant to the all of the FDA-approved CDK4/6 inhibitors (i.e., palbociclib, ribociclib, and abemaciclib) (**Figures 1C, D**, and **Supplementary Figures 1A, B**). The resistant clones divided under CDK4/6 inhibitor treatments with varied rates and doubling times, which suggests heterogeneous behavior among the cells (**Figures 1E, F**). We then explored the mechanisms of drug resistance in 12 randomly selected KKU-055R clones by immunoblotting (**Supplementary Figure 1C**). Virtually all of the representative clones demonstrated altered levels of proteins previously reported to be associated with CDK4/6 inhibitor resistance (21). More specifically, clones 8, 18, and 30 showed pRB downregulation; clones 12, 15, 18, 27, 28, and 29 showed CDK4-Cyclin Ds downregulation and cyclin E upregulation; and, clones 12, 15, 27, 28, and 30 showed CDK6 overexpression that correlated with slight upregulation of phospho-S6 kinase. These results indicated that we had established CDK4/6 inhibitor-resistant CCA clones that had developed varied adaptive molecular profiles to survive under CDK4/6 inhibitor treatment.

### Acquired Vulnerability Screening Uncovered Hypersensitivity of CDK4/6 Inhibitor-Resistant Clones to Ribosome Biogenesis Stress

**Figure 2:**
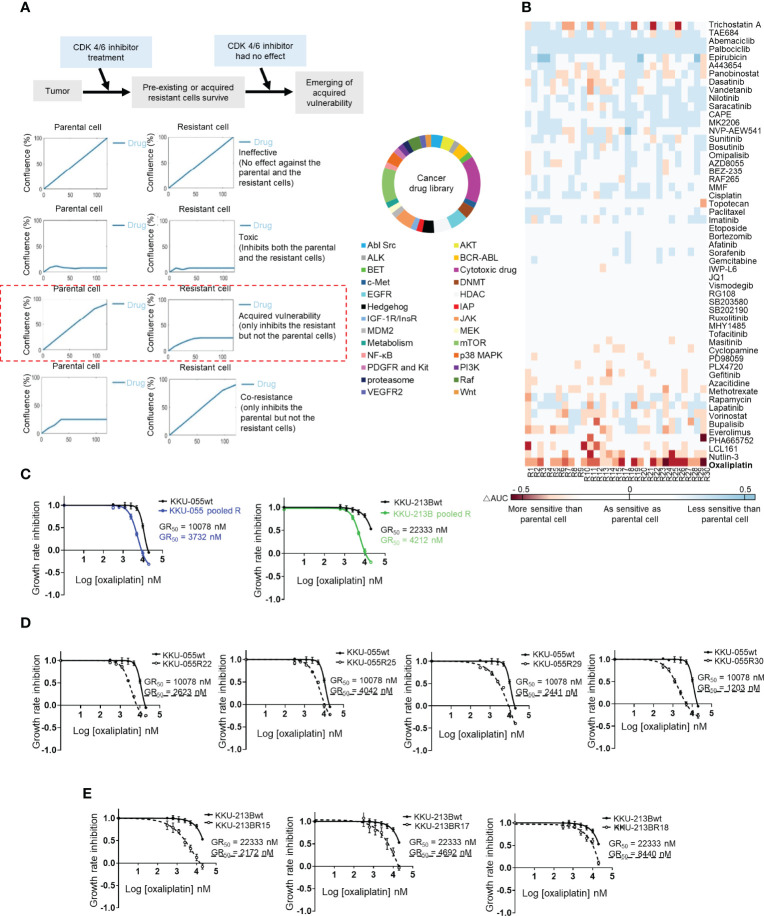
Cancer drug library screening reveal the acquired vulnerability of CDK4/6 inhibitor-resistant clones to ribosome biogenesis stress. **(A)** Acquired vulnerability concept (top). Drugs were considered to be acquired vulnerability-positive when the 5-day area under the cell proliferation curve (AUC) of a resistant clone was lower than 50% of the AUC of the parental cell (left). A small cancer drug library (right). **(B)** Heat map showing the relative sensitivity of each KKU-055-resistant clone compared to KKU-055wt (less sensitive: blue, more sensitive: red). **(C)** Oxaliplatin dose-response curves for KKU-055wt, KKU-213B, and their pooled resistant cells. **(D)** Oxaliplatin dose-response curves for KKU-055wt and resistant clones. **(E)** Oxaliplatin dose-response curves for KKU-213Bwt and resistant clones.

We used the IncuCyte^®^ Live-Cell Analysis System to perform acquired vulnerability screening by comparing the 5-day growth curves of twenty-nine resistant KKU-055R clones to the growth curves of KKU-055wt while under treatment with individual drugs from a small cancer drug library that covered 25 cancer pathways plus commonly used chemotherapies. We defined acquired vulnerability as positive when the average (n=4) area under the curve (AUC) of a resistant clone was smaller than the AUC of the parental cell by at least two fold (**Figure 2A**). As expected, we found all of the KKU-055R clones to universally resistant to all 3 of the CDK4/6 inhibitors (i.e., palbociclib, ribociclib, and abemaciclib) [**Figure 2B** (blue color indicates less sensitivity to a drug compared to the sensitivity observed in KKU-055wt]. None of the resistant clones acquired vulnerability to the standard first-line CCA chemotherapy gemcitabine or cisplatin, except clone 12, which developed sensitivity to cisplatin (shown in red). We also found that several resistant clones became hypersensitive to mTOR inhibitors [i.e., everolimus (13/29 clones), and rapamycin (6/29 clones)] (**Figure 2B**), which is consistent with previous findings from breast cancer studies that investigated whether targeting activated PI3K/mTOR signaling may overcome acquired resistance to CDK4/6-based therapies (21–23). Unexpectedly, we found that most of the resistant clones (25/29 clones, 86.2%) had acquired sensitivity to oxaliplatin, which is a platinum agent that is used in combination with fluoropyrimidine, or gemcitabine as one of the standard second-line CCA treatment (**Figure 2B**, **Supplementary Figure 2A**). To validate the acquired vulnerability to oxaliplatin, we generated oxaliplatin dose-response curves for several KKU-055R and KKU-213BR clones, as well as for pools of KKU-055R, and KKU-213BR cells. We found a consistent reduction in oxaliplatin GR50s in all the clones and pooled resistant cells (**Figures 2C–E**). Oxaliplatin is an atypical platinum agent, which unlike the prototypical cisplatin, kills cancer cells by interfering with ribosome biogenesis (24). Clonogenic survival assay confirmed that the resistant clones were more sensitive to oxaliplatin compared to KKU-055wt cells (**Supplementary Figure 2B**). We then tested the relative sensitivity of the resistant clones to other drugs known to interfere with ribosomal biogenesis, such as phenanthriplatin (24) and actinomycin D. We found most of the KKU-055R and 213BR clones tested and the pooled resistant clones to be vulnerable to anti-ribosome biogenesis drugs (**Supplementary Figures 2C–H**). To further confirm the target of oxaliplatin in the ribosomal pathway, we also treated CCA cells with omacetaxine, which is a ribosome inhibitor whose anti-cancer function relies on intact ribosome function. We found that increasing the dose of oxaliplatin antagonized the anti-cancer activity of omacetaxine, which suggests that both drugs exploit a similar biological pathway to inhibit cancer cells (**Supplementary Figure 3A**). Of note, the observed antagonism was more noticeable in resistant KKU-055R cells compared to the parental cells, which suggests a more pronounced ribosomal dependency in the resistant cells. In addition, to exclude the possibility that resistant cells acquired a vulnerability to DNA damage-induced killing, we treated KKU-055R clones with various doses of cisplatin. We found that none of the resistant clones acquired sensitivity to cisplatin compared to the KKU-055wt (**Supplementary Figure 3B**). We, therefore, concluded that our acquired vulnerability screen had uncovered an acquired vulnerability to ribosomal biogenesis stress that may be targeted by oxaliplatin, an anti-ribosomal biogenesis drugs.

### Altered Ribosome Expressions in CDK4/6 Inhibitor-Resistant CCA Cells

**Figure 3:**
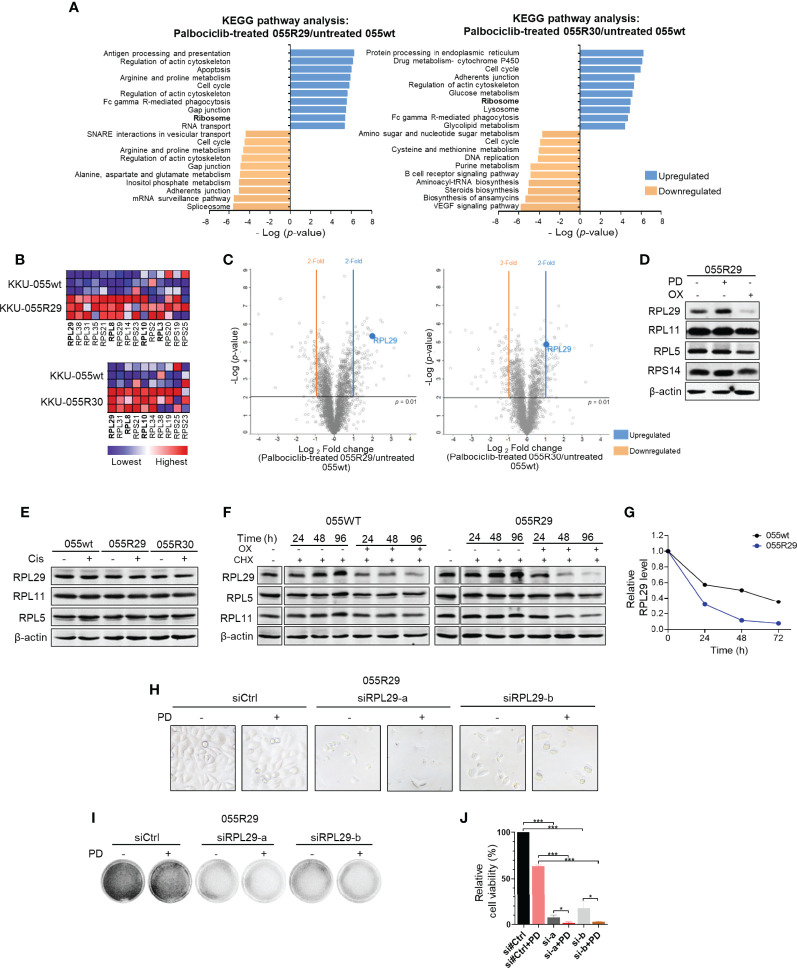
Dysregulated expression of specific ribosomal protein in CDK4/6 inhibitor-resistant CCA cells. **(A)** Kyoto Encyclopedia of Genes and Genomes (KEGG) pathway enrichments in palbociclib-treated KKU-055R29 (left) and KKU-055R30 (right) compared to untreated KKU-055wt. **(B)** Ribosome gene set enrichment shows significant ribosomal protein enrichment in palbociclib-treated KKU-055R29 and KKU-055R30 compared to untreated KKU-055wt (p<0.05). The heat map shows the enrichments in triplicate (highest in red, lowest in blue). Ribosomal proteins in the RPL29 complex are shown in bold. **(C)** Volcano plots of protein expressions in palbociclib-treated KKU-055R29 (left) and KKU-055R30 (right) compared to untreated KKU-055wt. **(D)** Western blot analysis of ribosomal proteins in KKU-055-resistant clone R29 after 24 hours of treatment with 1 µM palbociclib, 5 µM oxaliplatin, or vehicle. β-Actin was used as the loading control. **(E)** Western blot analysis of ribosomal proteins in KKU-055wt and KKU-055-resistant clones after 24 hours of treatment with 0.5 µM cisplatin or vehicle. **(F)** Western blot analysis of ribosomal proteins in KKU-055-resistant clone R29 (right) after 24-, 48-, and 96-hours of treatment with vehicle, 0.5 µM cycloheximide, or 0.5 µM cycloheximide combined with 5 µM oxaliplatin. **(G)** Quantitation of RPL29 half-life in KKU-055wt compared to that of KKU-055R. At each time point, the protein amount was quantitated and normalized relative to β-actin. **(H)** Phase-contrast images of KKU-055-resistant clone R29 treated with non-targeting siRNA (siCtrl) or RPL29 siRNAs (siRPL29-a, siRPL29-b) for 48 hours, and then treated with 0.5 uM palbociclib (PD) or vehicle for 72 hours. **(I)** Images of crystal violet staining from H. **(J)** Relative survival from F was quantified. The bars represent the average of 3 replicates ± standard deviation (SD). Analysis for statistical significance was performed using Student’s t-test (*p≤0.05, and ***p≤0.001).

To understand the acquired sensitivity to anti-ribosome biogenesis agents, we quantitatively analyzed the proteomic profiles of resistant clones. We selected KKU-055R30 with a low level of pRB, and KKU-055R29 with cyclin E overexpression to compare with KKU-055wt cells. We found quite similar proteomic changes in both resistant clones. Top changes that were shared between the two resistant clones were cell cycle (p=0.0000017 vs. p=0.0000013), amino acid and glucose metabolism (p=0.0000014 vs. p=0.0000085), phagocytosis (p=0.0000031 vs. p=0.000023), regulation of actin (p=0.00000078 vs. p=0.0000058), and ribosomal proteins (p=0.0000044 vs. p=0.000013), all respectively (**Figure 3A**). As a result of the acquired vulnerability to anti-ribosomal biogenesis agents observed in the resistant cells, we focused on common changes in ribosomal proteins. We found significant enrichment of 15, and 10 ribosomal proteins in KKU-055R29, and KKU-055R30, respectively (**Figure 3B**). Individually, Ribosomal Protein (RP) Large subunit 29 (RPL29) was the most significantly upregulated in both resistant cells compared to KKU-055wt (**Figure 3C**).

Interestingly, we found the levels of RPL29 in resistant cells to be associated with drug treatment. More specifically, RPL29 was upregulated under palbociclib treatment, but it was downregulated under oxaliplatin treatment (**Figure 3D** and **Supplementary Figures 4A, B**). This finding suggests a functional role of RPL29 in CCA survival under these two treatments. Interestingly, the level of RPL29 was also suppressed in oxaliplatin treatment in spite of palbociclib treatment (**Supplementary Figure 4B**). The drug-induced ribosomal changes were specific to RPL29, but not to the other key RPs in the ribosome checkpoint pathway, such as RPL5, RPL11, and RPS14 (**Figure 3D**). Of note, cisplatin treatment did not cause any noticeable change in RPL29 expression (**Figure 3E**). Interestingly, we also observed drug-induced changes in RPL29 in the CDK4/6 inhibitor sensitive KKU-055wt cells, in a lesser extent (**Supplementary Figure 4B**), which suggests that drug-induced changes in RPL29 are a consequence of molecular reprofiling in resistant cells. RPL29 is a long half-life protein that was stable for 96 hours under cycloheximide treatment. We found that oxaliplatin significantly shortened the half-life of RPL29 to 20 hours (**Figures 3F, G**). Oxaliplatin had no effect on the half-life of RPL5 or RPL11 (**Figure 5F**). Therefore, to survive CDK4/6 inhibition, CCA cells reprofiled their molecular networks as reflected by drug-induced changes in RPL29. To further investigate whether RPL29 plays an essential role in the survival of the resistant clones, we partially depleted RPL29 by 2 independent sequences of RPL29 siRNA (**Supplementary Figure 4C**). We found that RPL29 siRNA-a, and b, which depleted 50% of the endogenous RPL29, significantly decreased the survival of the resistant clones, and the addition of palbociclib further decreased the survival of the resistant clones (**Figures 3H-J**).

### Ribosome Biogenesis Checkpoint-Mediated Activation of p53 in CDK4/6 Inhibitor-Resistant Clones Under Oxaliplatin Treatment

Ribosomal biogenesis is tightly linked to cellular activities, such as growth and cell cycle progression. Perturbation of ribosomal biogenesis can cause nucleolar stress. The process through which RPs transmit nucleolar stress signals *via* MDM2-p53 has been described as a crucial tumor suppression mechanism (25). Downregulation of RPL29 has been linked to p53 activation by RPL5/11 sequestration of MDM2 in the nucleus (18, 26–28). Since we found that oxaliplatin promotes downregulation of RPL29, we hypothesized that oxaliplatin triggers RPL5/11-mediated MDM2 sequestration and p53 activation in CDK4/6 inhibitor-resistant cells.

**Figure 4:**
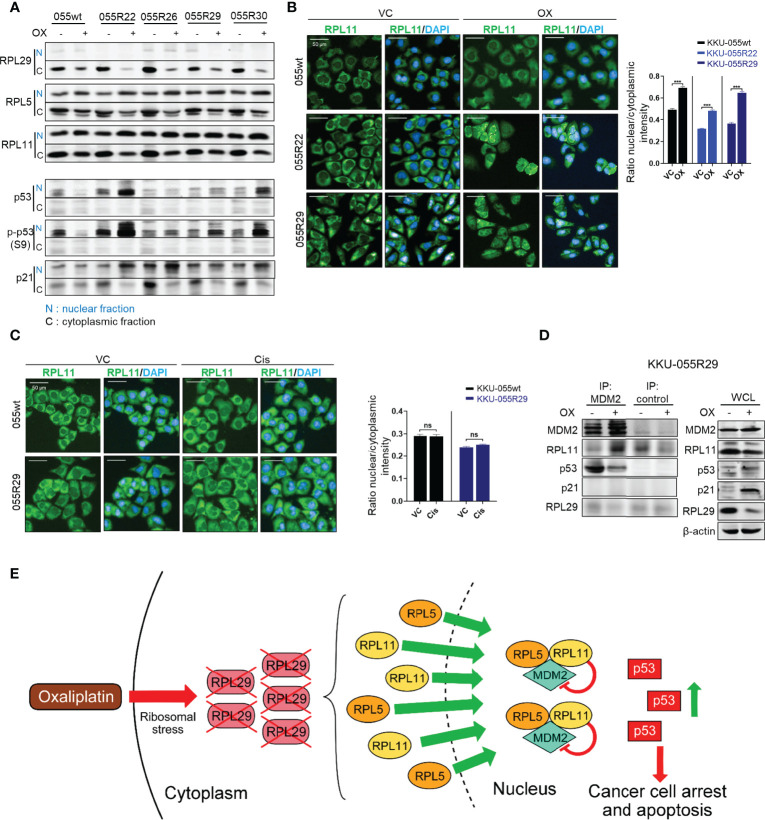
Oxaliplatin treatment promotes growth inhibition in the CDK4/6 resistant clones through p53 activation. **(A)** Western blot analysis of RPL29, RPL5, RPL11, p-p53 (S9), and p21 in the nucleus (N) and cytoplasm **(C)** of KKU-055wt and KKU-055-resistant clones after 24-hours of treatment with 5 µM oxaliplatin or vehicle. **(B, C)** Fluorescence images of RPL11 in KKU-055wt and KKU-055-resistant clones after 24 hours of treatment with 5 µM oxaliplatin (B) or 0.5 µM cisplatin **(C)** compared to vehicle control (VC). DAPI (4′,6-diamidino-2-phenylindole) staining was performed to identify the nucleus. Percent intensity quantification is shown in bar graphs. The bars represent the averages of 3 replicates ± SD. Analysis for statistical significance was performed using Student’s t-test (***p≤0.001, ns; not statistically significant). **(D)** Immunoprecipitation of MDM2 interacting proteins in a resistant clone under 24-hours of oxaliplatin treatment. Western blot analysis of the MDM2 interactors is shown on the left, and expressions of endogenous proteins are shown on the right. β-Actin was used as the loading control. **(E)** Schematic of oxaliplatin-induced RPL29 degradation and RPL5/11-MDM2-mediated p53 activation in palbociclib-resistant cells.

As shown by the immunoblots in **Figure 4A**, oxaliplatin downregulated RPL29 in all of the resistant clones tested (to a lesser extent in wild-type cells), and promoted relocation of RPL5 and RPL11 from the cytoplasm to the nucleus. We also detected activation of p53 as indicated by elevated p53 S9 phosphorylation, and upregulation of the p53 functional target p21 (**Figure 4A**). Translocation of RPL5 and RPL11 was also confirmed by immunofluorescence staining (**Figure 4B** and **Supplementary Figure 5A**). The drug-induced relocalizations of RPL5 and RPL11 were specific to oxaliplatin – not cisplatin (**Figure 4C** and **Supplementary Figure 5B**).

We demonstrated by co-immunoprecipitation an increase in MDM2-RPL5/11 complex in the resistant cells upon oxaliplatin treatment (**Figure 4D** and **Supplementary Figure 5C**). We also observed the departure of p53 from the MDM2 complex upon oxaliplatin treatment (**Figure 4D**). We, therefore, concluded that under oxaliplatin treatment, RPL29 was suppressed, and RPL5/11 were relocalized to the nucleus to sequestrate MDM2, which caused p53-mediated growth inhibition in the resistant cells (**Figure 4E**).

### Oxaliplatin Synergizes With Palbociclib to Inhibit CDK4/6 Inhibitor-Resistant CCA

**Figure 5:**
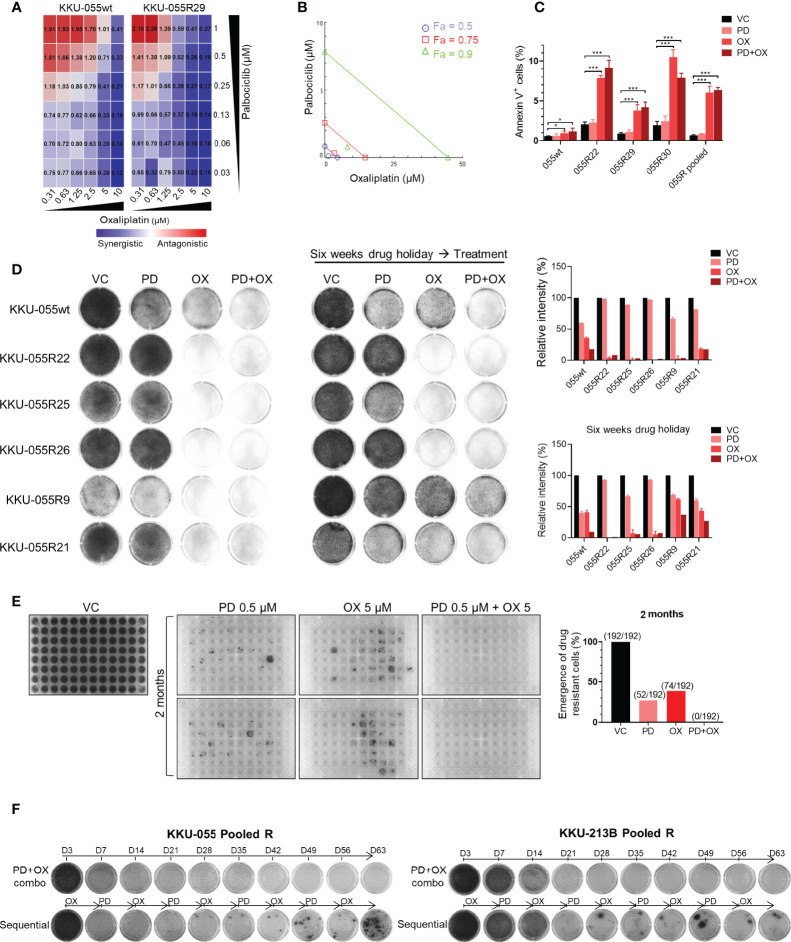
Dual oxaliplatin plus palbociclib synergistically inhibited emergence of drug-resistant cells. **(A)** Combination index matrices of indicated doses of palbociclib and oxaliplatin combination treatment in KKU-055wt and resistant clone R29. Colors in the matrix indicate different levels of drug effect (synergistic: blue, antagonistic: red). **(B)** Isobologram of 0.13 µM palbociclib and 1.25 µM oxaliplatin combination treatment in KKU-055 resistant clone R29. **(C)** Annexin V assay of the KKU-055wt and resistant clones treated with 0.13 µM palbociclib, 4 µM oxaliplatin, the combination of both, or vehicle for 48 hours. Percent annexin V-positive cells quantification is shown in bar graphs. The bars represent the average of 4 replicates ± SD. Analysis for statistical significance was performed using Student’s *t*-test (*p ≤ 0.05, **p ≤ 0.01, and ***p ≤ 0.001). **(D)** Clonogenic survival assay of KKU-055wt and resistant clones that were treated with 1 µM palbociclib, 5 µM oxaliplatin, the combination of both, or vehicle (left). The resistant clones were cultured in medium without drug for six weeks (drug holiday), and then treated with 1 µM palbociclib, 5 µM oxaliplatin, the combination of both, or vehicle (middle). The results are representative wells of triplicates. Percent intensity quantification is shown in bar graphs (right), and the bars represent the average of 3 replicates ± SD. **(E)** The emergence of drug-resistant cells was demonstrated by crystal violet staining. Two-month cultures of KKU-055wt cells under 0.5 µM palbociclib, 5 µM oxaliplatin, and the combination of both in 2 of 96-well plates (192 wells). The number of emergences well was counted and plotted in a bar graph (right). **(F)** KKU-055 pooled R and KKU-213B pooled R were treated with the combination of 0.5 µM palbociclib and 5 µM oxaliplatin (upper), or sequentially treated with 5 µM Oxaliplatin for 7 days and followed by 0.5 µM Palbociclib for another 7 days (lower) for 2 months. The emergence of the drug-resistant cell were shown in crystal violet staining.

We set forth to evaluate the efficacy of CDK4/6 inhibitor and oxaliplatin combination therapy for preventing cancer drug resistance. We performed *in vitro* cancer inhibition assays of different oxaliplatin+palbociclib combinations, and we calculated the combination index (CI) for each combination. We found that most of the evaluated combinations generated synergistic effect (CI <1), and that a higher dose of oxaliplatin produced stronger synergism (**Figure 5A**). Similar results were observed in the phenanthipatin+palbociclib combination (**Supplementary Figure 6A**). Results from isobologram confirmed the synergistic effect between oxaliplatin and palbociclib with 90%, 75%, and 50% growth inhibition (Fa 90, 75, 50, respectively) (**Figure 5B**). These results were also validated in 3D spheroid models (**Supplementary Figure 6B**).

Mechanistically, palbociclib exerts a cytostatic effect *in vitro* (**Figure 5C**). The addition of low-dose oxaliplatin (GR50 of parental KKU-055wt cells) effectuated small upregulation of the dying annexin V-positive KKU-055wt cells. In contrast, it caused significant increases in annexin V-positive cells in all resistant clones, as well as in the pooled cells. Similar results were observed in the oxaliplatin + plabociclib combination (**Figure 5C**).

Drug resistance, whether preexisting or acquired, is largely thought to be a stable and heritable process. However, over the past few decades, clinical evidence has suggested the role of unstable (reversible) non-heritable mechanisms of acquired drug resistance that affect chemotherapy and targeted agents. We, therefore, examined whether oxaliplatin can overcome both stable and reversible types of CDK4/6 inhibitor resistance. After a 6-week drug holiday, several CDK4/6 inhibitor-resistant clones (i.e., 22, 25, and 26) retained their CDK4/6 inhibitor resistance, which indicated stable resistance in these clones. In contrast, the resistance to palbociclib was reversed in some clones, such as 9 and 21 (**Figure 5D**), which indicates reversible resistance.

We found oxaliplatin to be effective against both stable and reversible resistance when given to the cells without drug holiday, or when given in combination with palbociclib (**Figure 5D**, left panel). After a 6-week drug holiday, oxaliplatin was still effective against the clones with stable resistance (clones 22, 25, 26). However, oxaliplatin monotherapy was not effective against the cells with reversible resistance. Interestingly, only the oxaliplatin+palbociclib combination was relatively effective against the reversed clones (**Figure 5D**, right panel). These findings indicate that continuous pressure from CDK4/6 inhibition is required to maintain acquired vulnerability to oxaliplatin. We then investigated the application of CDK4/6 inhibitor and oxaliplatin combination for preventing the emergence of drug-resistant cells. We performed long-term cultures of KKU-055wt cells under high doses of palbociclib, oxaliplatin, and the combination of the two for 2 months in 2 of 96-well plates (192 wells) (**Figure 5E**). Within 2 months, we found that 52 (27.1%) and 74 (38.5%) colonies of drug-resistant cells emerged in palbociclib and oxaliplatin monotherapy, respectively; however, there was no emergence (0%) of resistant cells from the combination treatment, which indicates a complete block of drug resistance by this drug combination. In contrast, complete suppression of resistant cells was not achieved by the palbociclib+cisplatin combination (**Supplementary Figure 6C**). Lastly, we found that the combination regimen was more effective compared to the sequential regimen in suppression of the pool drug resistant KKU-055R, and KKU-214BR cells (**Figure 5F**).

### Effect of Oxaliplatin+Palbociclib Combination Treatment in an *In Vivo* Model for CDK4/6 Inhibitor-Resistant CCA and Patient-Derived Organoid Models

**Figure 6:**
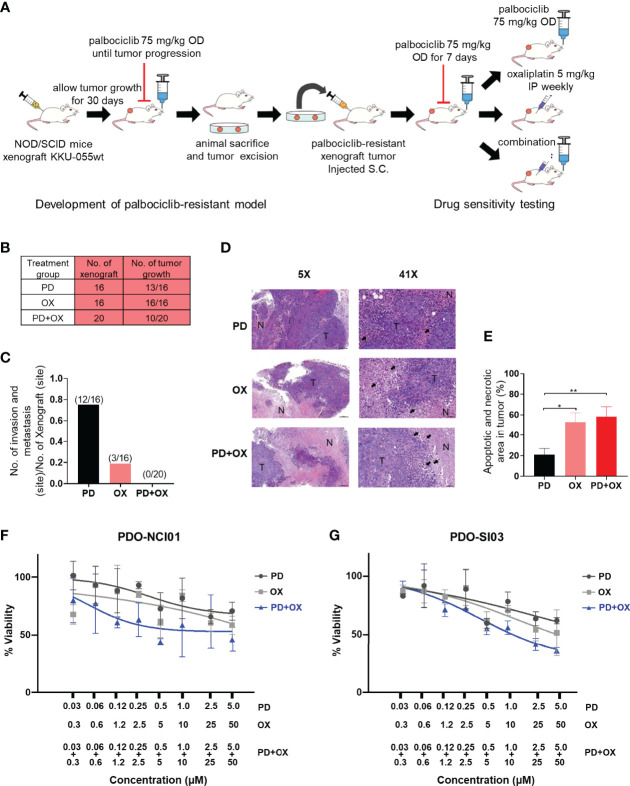
Palbociclib treatment enhances the antitumor effect of oxaliplatin in an in vivo model. **(A)** Schematic of the in vivo study. **(B)** The number of drug-resistant tumors that developed under Palbociclib (PD), oxaliplatin (OX), or palbociclib and oxaliplatin combination (PD+OX) treatment. **(C)** The number of invasion and metastasis sites in mice treated with palbociclib, oxaliplatin, or palbociclib and oxaliplatin combination treatment. **(D)** Hematoxylin and eosin (H&E) staining was used to identify areas of necrosis/apoptosis. (tumor area: T, coagulative necrosis area: N, apoptotic cell: black arrow). **(E)** Percent apoptotic and necrotic areas in each tumor were calculated and are shown in a bar graph. The bars represent the average of each treatment ± standard error of the mean (SEM). Analysis for statistical significance was performed using Student’s t-test (*p≤0.05, and **p≤0.01). **(F–G)** Dose response curves of patient-derived CCA organoids (PDO). The PDOs were treated with Palbociclib, Oxaliplatin, or combination of Palbociclib+Oxaliplatin as indicated. Error bars represent standard deviation of triplicate cultures.

To assess the physiological effect and relevance of the oxaliplatin+palbociclib combination, we developed a mouse xenograft model for CDK4/6 inhibitor-resistant CCA. CDK4/6 inhibitor-sensitive KKU-055wt cells were implanted into the flanks of NOS/SCID mice, and the tumor was allowed to grow to 0.5 cm in diameter. To obtain a CDK4/6 inhibitor-resistant tumor, we treated the mice with 75 mg/kg/day palbociclib until the tumor regrew under the treatment. The CDK4/6 inhibitor-resistant tumor was then engrafted into a cohort of NOD/SCID mice to generate the CDK4/6 inhibitor-resistant model (**Figure 6A**). Although 13/16 (81.3%) and 16/16 (100%) of the drug-resistant tumors developed under palbociclib monotherapy and oxaliplatin monotherapy, respectively, only half (10/20, 50%) of the palbociclib-resistant tumors grew under the oxaliplatin+palbociclib combination treatment, which suggests that the combination therapy was more effective than monotherapy in the *in vivo* setting (**Figure 6B**). This combination treatment was also effective in suppressing tumor invasion and metastasis. We did not detect any tumor metastasis or invasion in the mice under combination therapy (0/20 tumors injected). In contrast, we found 12/16 and 3/16 metastasis sites/tumor injected in mice treated with palbociclib and oxaliplatin monotherapy, respectively (**Figure 6C**). In agreement with the *in vitro* cell death results, tumors extracted from combination treated mice and oxaliplatin monotherapy treated mice contained a large proportion of cancer cell death and necrosis (**Figures 6D, E**). To evaluate the clinical relevance of the combination, we tested it against 2 models of drug-naïve patient-derived intrahepatic CCA organoid, and we found that the combination was effective against the CCA organoids and lowered of the effective dose of each drug (**Figures 6F, G**). Taken together, these results indicate the comparative effectiveness of oxaliplatin+palbociclib combination therapy against drug-naïve CCA and CDK4/6 inhibitor-resistant CCA cells in the *in vivo* settings.

## Discussion

Acquired vulnerability screening in CCA clones under CDK4/6 inhibitor treatment revealed that while attempting to survive and proliferate under CDK4/6 inhibition, CCA cells alter their ribosomal balance, which results in a novel drugable weakness. Our approach did not focus on identification of the drug-resistant mechanism, but rather on the exploitable profile of the resistant cells. The resistant clones that we developed reflected clonal heterogeneity, and possessed several possible CDK4/6 inhibitor-resistant mechanisms. Fortunately, we found that almost all of them succumbed to the anti-ribosome biogenesis drug oxaliplatin. *Via* a series of experiments, we demonstrated that different CDK4/6 inhibitor-resistant mechanisms may translate into a common weakness in ribosome biogenesis that can be targeted by oxaliplatin, one of the standard drug used in CCA treatment.

In addition to their primary function in ribosome biogenesis, many RPs have extraribosomal functions (22, 23), such as apoptosis, cell cycle arrest, cell migration, and invasion (24). RPL29 was identified as an oncoprotein because of its ability to promote cancer cell proliferation, to promote tumor angiogenesis, and to inhibit cell differentiation (25–28). Expression of RPL29 was also reported to be associated with cancer drug resistance (29). In this study, we showed that RPL29 is a central protein that enables the survival of CCA under CDK4/6 inhibition, and that oxaliplatin treatment results in early degradation of RPL29 and cancer cell death, which is mediated at least partly by p53-mediated cell death. A functional link between ribosome biogenesis stress and cell cycle control was recently established. Imbalanced expression of ribosomal proteins can trigger cell cycle arrest or cell death as a result of CDK4 binding and blocking by RPS14 or RPL22, or p53 activation by RPL5 or RPL11 (30–32). Interestingly, we showed a novel feedback mechanism by which cell cycle arrest by drug inhibitor stimulates upregulation of an oncogenic ribosomal protein. Further study is needed to elucidate how CDK4/6 inhibition causes RPL29 upregulation.

CDK4/6 inhibitors have shown great potential as new resources against cancer. However, their effect as single agents is limited, and the focus now is on identifying novel drug combination strategies. The cooperation between CDK4/6 inhibitors and endocrine therapy has been quite successful in estrogen-positive breast cancers. Other pathways that depend on CDK4-cyclin D complex, such as RAS-ERK and PI3K-AKT-mTOR, may also be good options for combination with CDK4/6 inhibition (33). Our results demonstrate a new approach in which we induced acquired dependency of CCA cells on ribosome biogenesis created by CDK4/6 inhibition. In agreement with this notion, our results showed that continuous pressure from CDK4/6 inhibition is needed to produce optimum anti-cancer activity. By way of example, we found that the oxaliplatin+palbociclib combination outperformed oxaliplatin monotherapy in resistant mouse model.

Since oxaliplatin is already a component of some of the standard regimens for CCA. i.e. FOLFOX, FOLFIRINOX, GemOx, the addition of a CDK4/6 inhibitor to oxaliplatin is an appealing possibility.

We showed that oxaliplatin treatment can accelerate RPL29 degradation, but we still do not know how RPL29 is upregulated by CDK4/6 inhibition, especially in CDK4/6 inhibitor-resistant cells. One possible explanation is that the activated PI3K-AKT-mTOR pathway in the resistant clones causes collateral overproduction of RPs (34). However, this cannot be a comprehensive explanation because only about half of the clones contained an activated PI3K-AKT-mTOR pathway. Disturbed ribosome biogenesis could also be the result of altered RB or p130 activities in a cell trying to survive under CDK4/6 inhibition (35) thereby causing derangement in rRNA expression. We also do not know whether CDK4/6 inhibition-induced acquired vulnerability to oxaliplatin also occurs in other types of cancer cells, or in cells with inherited resistance to CDK4/6 inhibition.

## Data Availability Statement

The raw data supporting the conclusions of this article will be made available by the authors, without undue reservation.

## Ethics Statement

The studies involving human participants were reviewed and approved by The Institutional Review Board for Human Research. The patients/participants provided their written informed consent to participate in this study. The animal study was reviewed and approved by The Faculty of Medicine, Siriraj Hospital Mahidol University – Institute Animal Care and Use Committee.

## Author Contributions

OS, SP, SA, BP, JM, MS performed the experiments KK, KC, RC, TP, SO, SS, SJ supervised and provided critical comments, experimental framework, and experimental design. RC, SO provided important resources and clinical specimens. OS, JM, TP, SS, SJ analyzed and interpreted the data. OS, SJ wrote and edited the manuscript. SJ provided financial support to the project. All authors contributed to the article and approved the submitted version.

## Funding

This study was funded by grants from the National Science and Technology Development Agency (NSTDA) of Thailand, the Japan Science and Technology Agency, and the National Institute of Allergy and Infectious Diseases of the United States as part of the e-ASIA Joint Research Program (e-ASIA JRP); the NSTDA (P-15-50208); the Thailand Research Fund (RSA5880038); the Siriraj Research Fund; the Foundation for Cancer Care, Siriraj Hospital; and, the Advanced Research in Pharmacology Fund, Siriraj Foundation (D003421). The aforementioned funding agencies had no influence on the interpretation of data, the final conclusions drawn, or the decision to publish this report.

## Conflict of Interest

The authors declare that the research was conducted in the absence of any commercial or financial relationships that could be construed as a potential conflict of interest.

## Publisher’s Note

All claims expressed in this article are solely those of the authors and do not necessarily represent those of their affiliated organizations, or those of the publisher, the editors and the reviewers. Any product that may be evaluated in this article, or claim that may be made by its manufacturer, is not guaranteed or endorsed by the publisher.
