# Supplementary material for: The Acquired Vulnerability Caused by CDK4/6 Inhibition Promotes Drug Synergism Between Oxaliplatin and Palbociclib in Cholangiocarcinoma
Source: Front Oncol. 2022 May 17;12:877194. doi: 10.3389/fonc.2022.877194 (PMC9157389; doi:10.3389/fonc.2022.877194)
Supplement: Supplementary file 1 [file DataSheet_1.pdf]

Supplementary figure 1

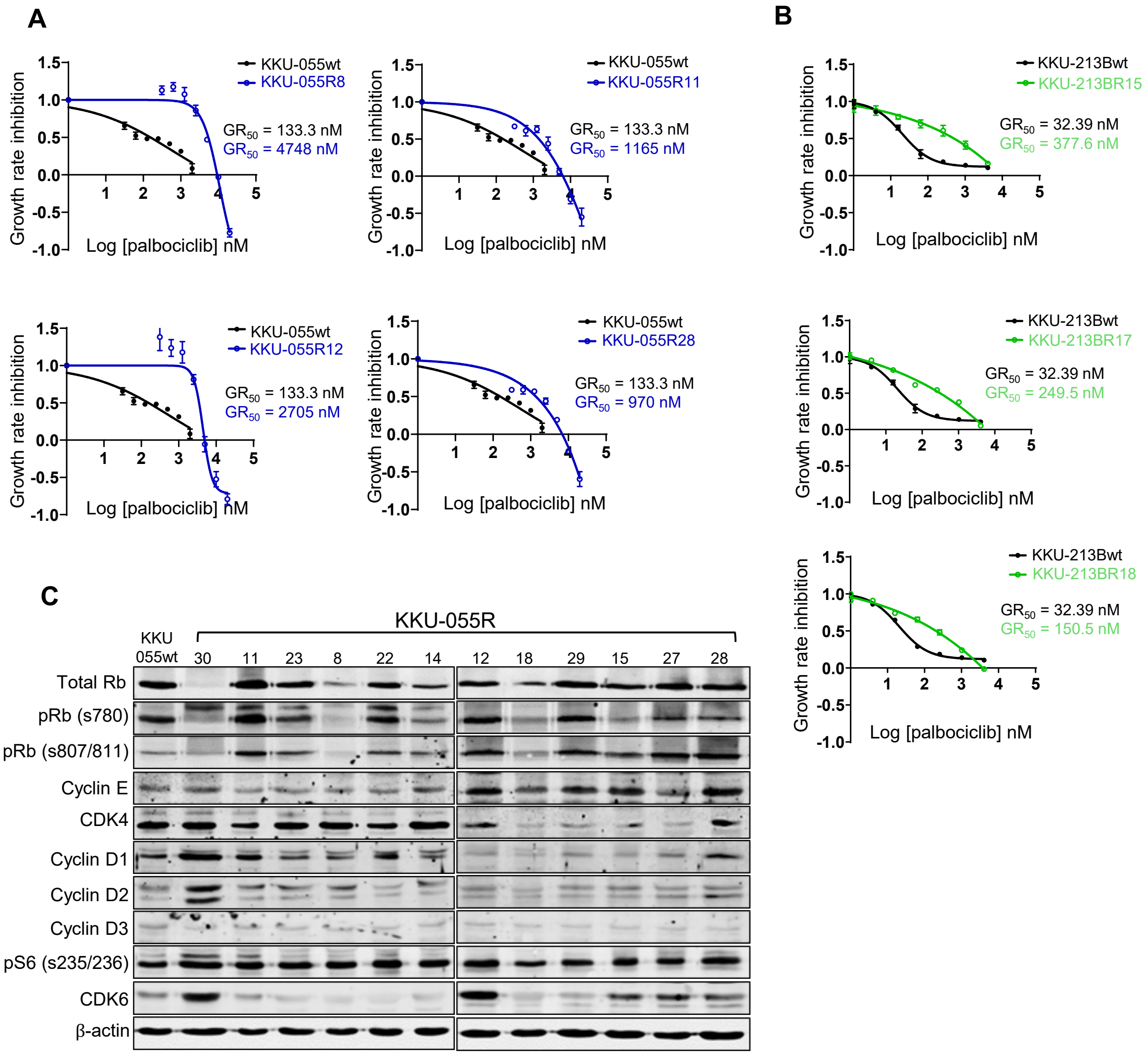

(**A-B**) Palbociclib dose-response curves of the KKU-055wt and KKU-055 resistant clones (A) and the KKU-213Bwt and KKU-213B resistant clones (B).
(**C**) Western blots of cell cycle proteins in the KKU-055wt and resistant clones.

Supplementary figure 2

A

Oxaliplatin	R24	R30	R22	R25	R26	R17	R19	R27	R12	R7	R11	R3	R15	R29	R1	R2	R20	R23	R8	R10	R6	R5	R28	R14	R13	R18	R9	R21	R4
Nutlin-3	R11	R25	R7	R17	R14	R27	R24	R1	R26	R15	R22	R13	R3	R29	R30	R9	R10	R6	R20	R5	R8	R28	R12	R18	R23	R21	R2	R4	R19
Everolimus	R5	R1	R13	R24	R25	R20	R30	R7	R21	R6	R3	R14	R2	R15	R10	R17	R11	R9	R12	R28	R22	R27	R4	R8	R29	R23	R19	R18	R26
Vorinostat	R7	R29	R10	R15	R30	R24	R6	R11	R21	R13	R12	R1	R17	R19	R26	R25	R5	R27	R14	R3	R20	R22	R4	R9	R18	R8	R23	R28	R2
LCL161	R1	R10	R12	R24	R11	R3	R5	R13	R21	R27	R28	R25	R17	R20	R7	R19	R22	R14	R8	R9	R26	R2	R23	R30	R6	R15	R4	R29	R18
Lapatinib	R19	R11	R1	R7	R22	R24	R13	R27	R14	R8	R9	R26	R2	R17	R23	R5	R25	R20	R30	R21	R6	R3	R15	R10	R12	R28	R4	R29	R18
Methotexate	R24	R27	R1	R30	R6	R20	R9	R8	R21	R2	R17	R5	R14	R23	R7	R15	R12	R13	R11	R28	R4	R3	R25	R10	R19	R22	R26	R29	R18
Rapamycin	R1	R20	R13	R6	R11	R24	R14	R21	R3	R9	R15	R25	R17	R10	R28	R12	R27	R30	R8	R4	R29	R19	R23	R7	R26	R22	R5	R18	R2
Trichostatin	R26	R22	R19	R7	R11	R8	R25	R13	R1	R20	R24	R9	R27	R29	R17	R5	R30	R15	R3	R4	R28	R14	R2	R6	R21	R23	R10	R12	R18
Bupalisib	R12	R28	R10	R30	R1	R25	R5	R2	R20	R11	R14	R15	R24	R21	R7	R13	R6	R3	R4	R22	R27	R9	R17	R19	R8	R18	R23	R26	R29
MK2206	R14	R17	R9	R7	R30	R15	R12	R8	R21	R13	R11	R20	R2	R28	R24	R5	R1	R4	R3	R23	R27	R25	R10	R19	R22	R26	R6	R29	R18
Vandetanib	R11	R5	R20	R14	R13	R9	R1	R22	R4	R7	R3	R15	R17	R21	R6	R27	R23	R26	R12	R24	R19	R8	R25	R29	R30	R28	R2	R10	R18
Sunitinib	R22	R26	R8	R14	R20	R27	R4	R17	R9	R6	R1	R7	R19	R21	R28	R23	R13	R15	R11	R24	R3	R25	R10	R12	R30	R29	R5	R18	R2
PHA665752	R30	R11	R14	R3	R4	R5	R6	R7	R8	R9	R10	R1	R12	R13	R2	R15	R17	R18	R19	R20	R21	R22	R23	R24	R25	R26	R27	R28	R29
Topotecan	R30	R1	R2	R3	R4	R5	R6	R7	R8	R9	R10	R11	R12	R13	R14	R15	R17	R18	R19	R20	R21	R22	R23	R24	R25	R26	R27	R28	R29

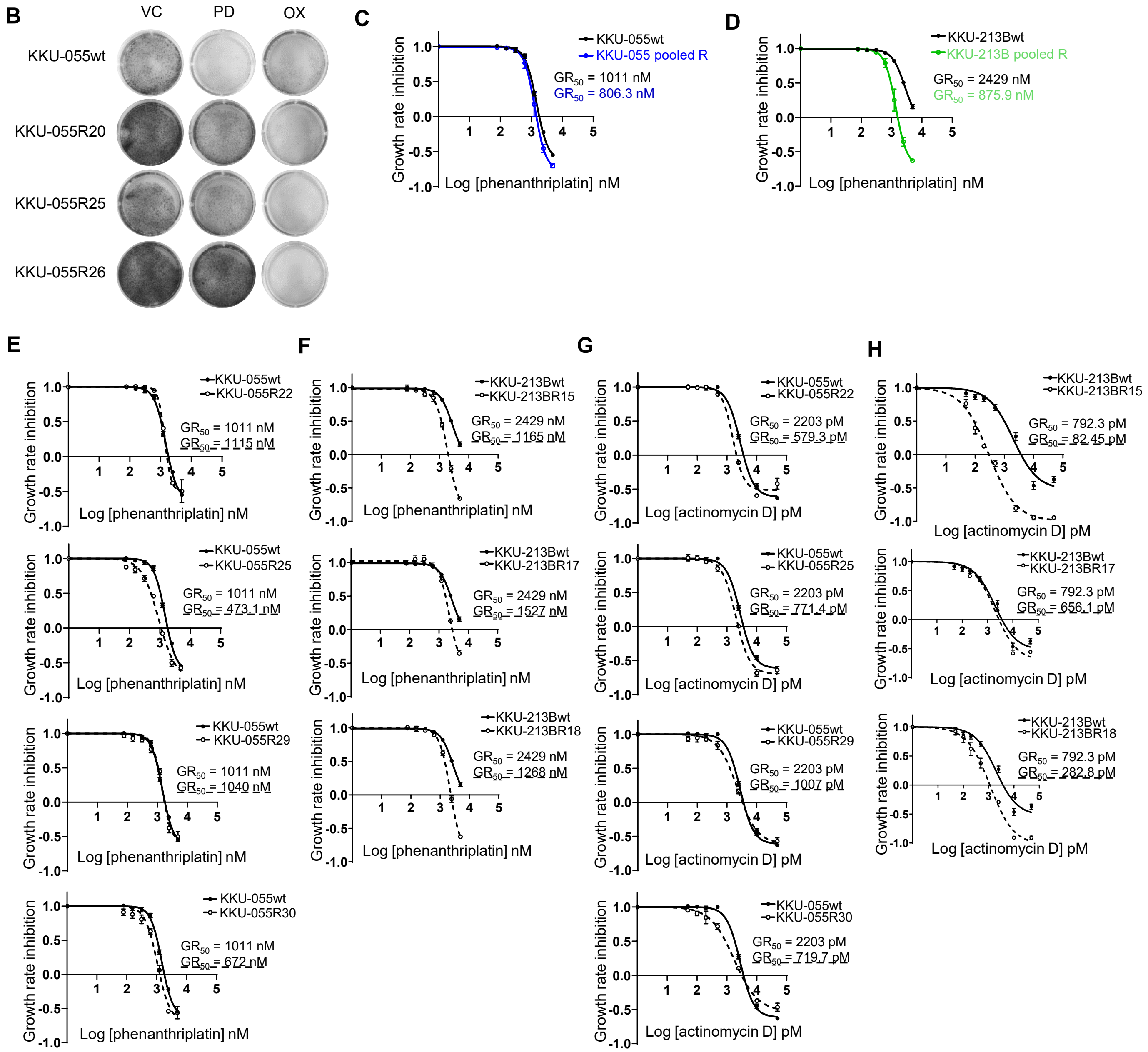

(**A**) Top 15 drugs which more effective in KKU-055 resistant clones compared to the parental counterpart. Clone numbers shown in red. The clones which were less sensitive were shown in yellow. Oxaliplatin was effective in 25 out of 29 resistant clones.
(**B**) Clonogenic survival assay of KKU-055wt and resistant clones treated with Palbociclib, Oxaliplatin, or vehicle. Images show representative wells of triplicates.
(**C-D**) Phenanthriplatin dose-response curves of the KKU-055wt (C), KKU-213Bwt (D), and pooled resistant cells.
(**E**) Phenanthriplatin dose-response curves for KKU-055wt and resistant clones.
(**F**) Phenanthriplatin dose-response curves for KKU-213Bwt and resistant clones.
(**G**) Actinomycin D dose-response curves for KKU-055wt and resistant clones.
(**H**) Actinomycin D dose-response curves for KKU-213Bwt and resistant clones.
Error bars represent standard deviation of triplicate cultures.

Supplementary figure 3

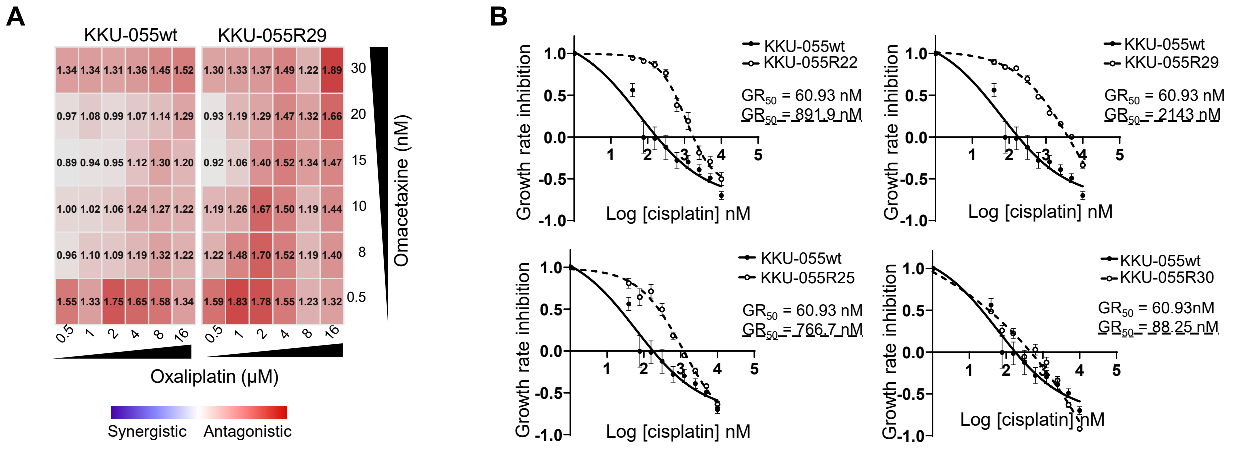

(**A**) Combination index matrices of indicated doses Omacetaxine and Oxaliplatin combination pairs in the KKU-055wt and resistant clone R29. Colors in the matrix indicate different levels of drug effect (synergistic appeared in blue and antagonistic effect appeared in red).

(**B**) Cisplatin dose-response curves of KKU-055wt and a resistant clones.

Supplementary figure 4

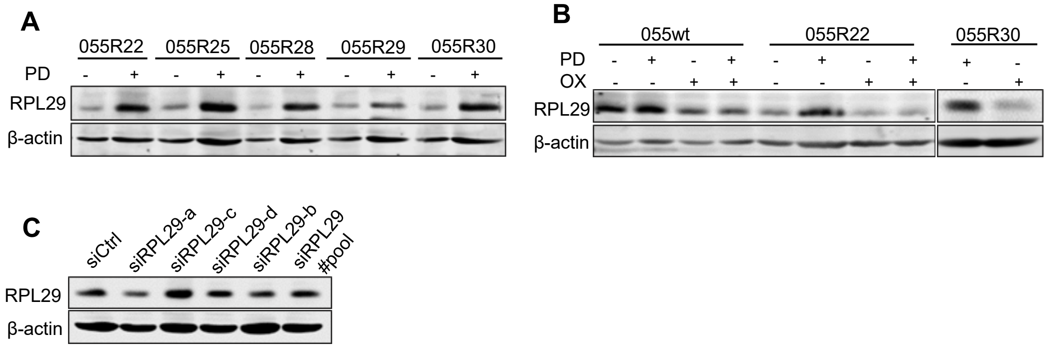

(**A**) Western blots of RPL29 KKU-055 resistant clones, with or without 1 μM Palbociclib treatment for 24 hours.

(**B**) Western blots of RPL29 in KKU-055wt, and resistant clones (R22, R30) under a vehicle, Palbociclib, Oxaliplatin, or combination treatment.

(**C**) Western blots of RPL29 expressions in the KKU-055 resistant clone R29 transfected with non-targeting siRNA (siCtrl) or RPL29 siRNA (siRPL29-a to d and pooled).

Supplementary figure 5

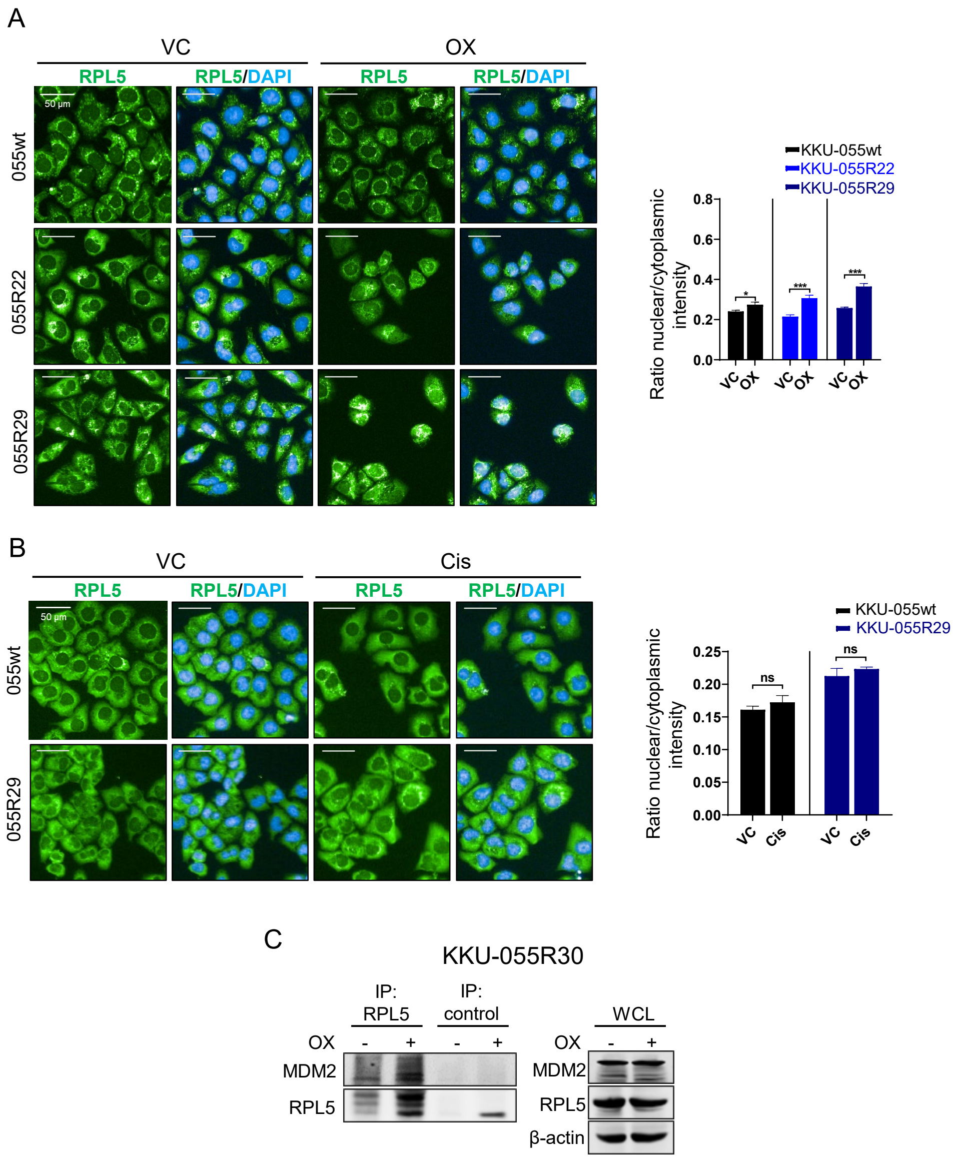

(**A-B**) Fluorescent images of RPL5 in the KKU-055wt and the KKU-055 resistant clones after 24 hours treatment with 5 µM Oxaliplatin (A), 0.5 µM cisplatin (B) or vehicle. DAPI was stained to identify the nucleus. Percent intensity was quantified in a bar graph. The bars represent the averages of 3 replicates ± SD. Statistical significances were analyzed by Student's t-test (**p* ≤0.05, ***p* ≤0.01, and ****p* ≤0.001).

(**C**) Western blots of KKU-055 resistant clone R30 after immunoprecipitation of RPL5 complexes (left), and whole-cell lysate (WCL) after 24 hours treatment with 5 µM Oxaliplatin or vehicle (right).

Supplementary figure 6

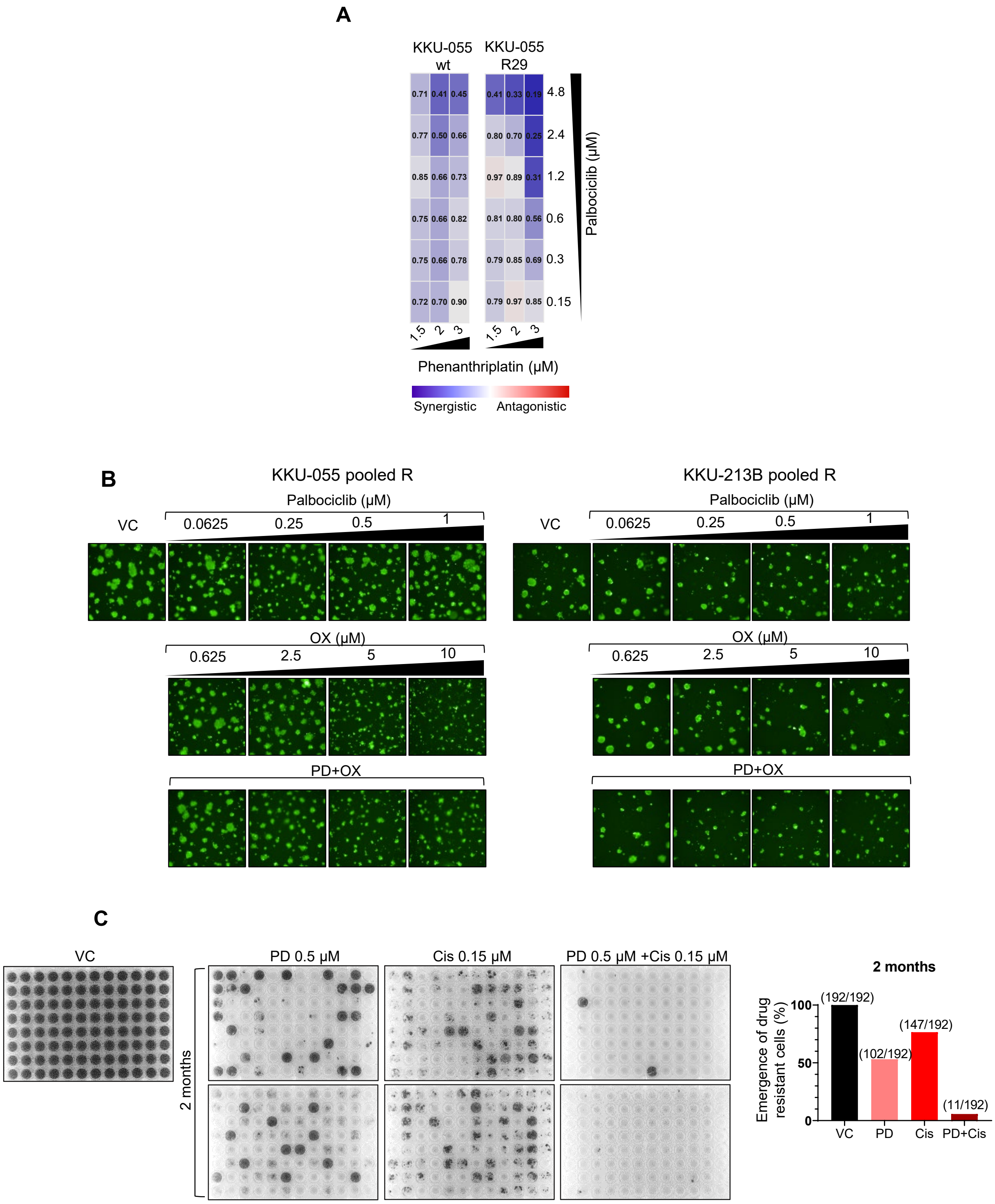

(**A**) Combination index matrices of indicated doses Palbociclib and phenanthriplatin combination treatment in the KKU-055wt and resistant clone R29. Colors in the matrix indicate different levels of drug effect (synergistic: blue, antagonistic: red).
(**B**) Images of the KKU-055 (pooled) resistant spheroids (left), the KKU-213B (pooled) resistant spheroids (right) treated with indicated doses of Palbociclib, Oxaliplatin, the combination, or vehicle. Images show representative wells of triplicates.
(**C**) The emergence of the drug-resistant cells was shown in crystal violet stained images. Two months cultures of KKU-055wt cells under 0.5 μM Palbociclib, 0.5 μM cisplatin, and the combination of both in 2 of 96-well plates (192 wells). the number of emergences well was count and plot in a bar graph (right)
