# Supplementary material for: The Acquired Vulnerability Caused by CDK4/6 Inhibition Promotes Drug Synergism Between Oxaliplatin and Palbociclib in Cholangiocarcinoma
Source: Front Oncol. 2022 May 17;12:877194. doi: 10.3389/fonc.2022.877194 (PMC9157389; doi:10.3389/fonc.2022.877194)
Supplement: Supplementary file 2 [file DataSheet_2.docx]

Supplementary Material

Materials and Methods

**Drug library screening**

The 55 cancer drugs used in this study were purchased from Selleck Chemicals. Cells were seeded in 384-well plates at a density of 1,000 cells/well and cultured for 24 hours. Cells were then treated with drugs at a GR75 dose of parental (wt) cells in quadruplicate using an epMotion Liquid Handling Workstation (Eppendorf, Hamburg, Germany). Dimethyl sulfoxide (DMSO) or dimethyl formamide (DMF) treatment was used as vehicle control. Cell proliferation was monitored using an IncuCyte® Live-Cell Analysis System (Sartorius Stedim Biotech, Göttingen, Germany) by capturing images every 12 hours for 120 hours. Data were analyzed using the integrated confluence algorithm of the occupied area (% confluence) of cell images over time. The area under the curve (AUC) was calculated.

**Drug synergism testing**

Five hundred cells were seeded into each well of 384-well plates. The data were analyzed based on the multiple drug effect equation of Chou-Talalay using CompuSyn software version 1.0 (ComboSyn, Inc., Paramus, NJ, USA). A combination index (CI) <1, =1, and >1 represent synergistic, additive, and antagonistic effects, respectively.

**Antibodies**

Rb (Cell Signaling Technology, Inc.,Danvers, MA, USA, #9309), pRb (s780)(Cell Signaling, USA, #8180), pRb (s807/811)(Cell Signaling, USA, #8516) pS6 (Cell Signaling, USA, #4858) p53 (Cell Signaling, USA, #48818) pp53 (s9) (Cell Signaling, USA, #9288), β-actin(Santa Cruz Biotechnology, Inc., Dallas, Tx, USA, SC-47778), cyclin D1 (Santa Cruz, USA, SC-753), cyclin D2 (Santa Cruz, USA, SC-593), cyclin D3 (Santa Cruz, USA, SC-182), cyclin E (Santa Cruz, USA, SC-481), CDK4 (Santa Cruz, USA, SC-260) CDK6 (EMD Millipore, Ca, USA, MABC 280), RPL5 (Abcam, Cambridge, UK, ab86863), RPL11 (Abcam, UK, ab79352), RPS14 (Abcam, UK, ab246916), RPL29 (Abcam, UK, ab88514) , MDM2 (Abcam, UK, ab16895).

**Apoptosis assay**

Annexin V apoptosis assay was performed by seeding 75,000 cells into 24-well plates. The next day, the culture medium was removed. The cells were washed with phosphate-buffered saline (PBS) and reincubated in new culture medium treated with 0.3 μM palbociclib, 4 μM oxaliplatin, or combination 0.3 μM palbociclib and 4 μM oxaliplatin for 48 hours. Apoptosis detection was performed using an Annexin V-FITC Apoptosis Detection Kit (Abcam, Cambridge, UK, ab14085) according to the manufacturer’s protocol. Four replicates were obtained for each drug treatment and each cell line. Data were analyzed using CytExpert software version 2.1.0.92 (Beckman Coulter Life Sciences, Indianapolis, IN, USA).

**Clonogenic survival assay and crystal violet staining**

Twenty-five thousand cells/well were seeded into six-well plates. Each cell line was incubated in complete media without any drug treatment for 0 or 6 weeks (for drug holiday). The next day, the culture medium was removed and new culture medium treated with 0.5 μM palbociclib, 5 μM oxaliplatin, or the combination of both was added into each well and reincubated for 5 days. The medium was removed at the end of the experiment after which the cells were washed in pre-cooled PBS, fixed in 10% neutral formalin, and stained with 0.5% crystal violet solution in 25% methanol. The cells were then washed with tap water until all stains were removed, air-dried, and photographed.

**Emergence of drug-resistant cells**

Parental KKU-055 cells (KKU-055wt) were plated in 96-well plates (Corning, Inc., Corning, NY, USA) in duplicates at 5,000 cell/well density. At 24 hours after seeding, a GR50 dose of drug-containing medium was added, and 0.5% DMSO was used as VC. Existing medium was replaced with fresh drug-containing medium every 5 days for 2 months after which the cells were fixed and stained following crystal violet staining protocol. For the sequential and combination treatment, the KKU-055 pooled R, and KKU-213B pooled R 100,000 cells/well were seeded in 6-well plates and maintained in 0.5 uM Palbociclib. At 72 hours after seeding, cells were treated with a combination of 0.5 µM palbociclib and 5 µM oxaliplatin (upper), or sequentially treated with 5 µM oxaliplatin for 7 days followed by 0.5 µM Palbociclib for 7 days (lower) for 2 months. New medium containing drugs were routinely refreshed every 3 days.

**Target gene silencing**

Small interfering RNAs (siRNAs) were transfected using RNAiMAX Reagent (Thermo Fisher Scientific) according to the manufacturer’s protocol. The target sequences for RPL29 genes were 5’AAGCGTGCTCGTGCC CGTATT-3’ (a)*,* 5’-TCCGGCGTTGTTGACCCTATT-3’ (b), 5’-CCTAAAGAAGATGCAGGCCAA3’ (c), and 5’-CTCCTGTGCTATTTGTACAAA-3’ (d) (Qiagen, Hilden, Germany). At 48 hours after siRPL29 transfection, the resistant clones were treated with 0.5 uM palbociclib for 72 hours. DMSO vehicle treatment was used as control.
